# Clinical and genetic analysis of atypical parathyroid adenoma compared with parathyroid carcinoma and benign lesions in a Chinese cohort

**DOI:** 10.3389/fendo.2023.1027598

**Published:** 2023-01-26

**Authors:** Yingyu Chen, An Song, Min Nie, Yan Jiang, Mei Li, Weibo Xia, Ou Wang, Xiaoping Xing

**Affiliations:** Department of Endocrinology, Key Laboratory of Endocrinology, National Health Commission, Peking Union Medical College Hospital, Chinese Academy of Medical Science & Peking Union Medical College, Beijing, China

**Keywords:** primary hyperparathyroidism, atypical parathyroid adenoma, parathyroid carcinoma, parathyroid adenoma, parathyroid hyperplasia, genetic analysis

## Abstract

**Context:**

The malignant potential and molecular signature of atypical parathyroid adenoma (APA) remain elusive. Data from Asia are still lacking.

**Design and setting:**

This was a retrospective study on a large APA cohort in a single center from mainland China.

**Methods:**

A total of 320 patients with primary hyperparathyroidism (PHPT), containing 79 APA, 79 Parathyroid cancer (PC) and 162 benign lesions cases, were enrolled after surgery for collection of clinical data and genetic analysis.

**Results:**

APA patients showed earlier mean onset age than benign group (46.9 ± 17.1 vs. 52.0 ± 14.3 yrs). Less bone involvement and gastrointestinal symptoms were presented in APA compared to PC (35.4% vs. 62.0%, and 17.7% vs. 41.8%), while more urolithiasis was seen in APA than in benign lesions (57.0% vs. 29.6%). The APA group had moderate hypercalcemia (mean 3.02 ± 0.44mmol/L) with elevated serum PTH (median 593.0pg/ml) and proportion of hypercalcemic crisis as 22.8%, all higher than those of benign lesions but lower than those of PC group. The recurrence/no remission rate of the APA group was significantly lower than that of the PC and similar to the benign group (5.1% vs. 31.6% vs. 3.1%). Germline CDC73 mutation was the most common molecular abnormality in both PC and APA subjects. APA patients with nonsynonymous germline variants showed earlier onset age (28.5 ± 16.9 vs. 48.1 ± 17.7 yrs) and more cases developing no remission/recurrence (25.0% vs. 0.0%).

**Conclusions:**

Patients with APA presented clinical and biochemical characteristics much less severe than PC and resembling the benign neoplasms, with a relatively good prognosis. Germline gene variations were associated with earlier onset and probably more recurrence of PHPT in APA.

## 1 Introduction

Primary hyperparathyroidism (PHPT) is the third most common endocrine disorder in western countries. In PHPT, primary abnormal parathyroid tumors inappropriately secret excessive parathyroid hormone (PTH) and thus lead to hypercalcaemia and a series of symptoms.

According to the existing data, almost all PHPT cases are caused by benign parathyroid lesions. Parathyroid adenoma (PA) is the most common type (~85%), with mostly single parathyroid gland being involved. Parathyroid hyperplasia (PH) arises in 10-15% PHPT patients (1, 2). Atypical parathyroid adenoma (APA) and parathyroid carcinoma (PC) occur rarer with PC being the rarest type (~1% in the western world and up to 8% in Asia) (3–5). Up to now, a total of less than 1, 000 cases of APA have been reported in different cohorts with only several studies including over 50 patients (6–11). Due to its rarity, there is still no general consensus on treatment and follow-up.

The term ‘APA’ or ‘atypical parathyroid neoplasm (APN)’ has been used to describe a subgroup of parathyroid tumors with uncertain malignant potential, having histological features suspicious for PC (i.e., solid growth pattern, prominent fibrous bands, questionable capsular invasion, adherence to surrounding tissues, marked cellular/nuclear atypia and increased mitotic figures) but lacking evidence of unequivocal invasion and/or metastasis which are the key morphological features of PC (12). It is sometimes difficult to make the differential diagnosis between APA and PC, which may be a big challenge even for an experienced pathologist. In some cases, the initial diagnosis of APA may be revised to carcinoma, because of the local reoccurrence or metastasis (13).

PHPT can be sporadic or familial/syndrome-related type, accounting for 90% and 10% of the disease, respectively. Familial PHPT consists of multiple endocrine neoplasia (MEN) type 1 (MEN1), type 2A (MEN2A), type 4 (MEN4) and hyperparathyroidism-jaw tumor syndrome (HPT-JT), caused by known germline genetic mutations (14). PC is commonly sporadic, but it may occur in familial/syndrome-related PHPT, including HPT-JT and, very rarely, MEN1 and MEN2A (15, 16). Up to 70% of sporadic PC carry a somatic mutation of the *CDC73* gene, with one-third of apparently sporadic PC having germline *CDC73* mutations. Our knowledge of the molecular pathogenesis of parathyroid carcinoma and benign adenoma has largely increased over the last decades. On the contrary, understanding of the molecular mechanisms underlying atypical parathyroid adenoma still lacks progress.

This study aimed to investigate the differences of clinical characteristics, biochemistry, outcomes and genetic features between APA and PC, in order to help construct treatment and follow-up strategies.

## 2 Subjects and methods

### 2.1 Subjects

From November 1992 to November 2019, 79 APA and 79 PC patients with relative complete medical records diagnosed and treated at Peking Union Medical College Hospital (PUMCH) were consecutively included in this study. A total of 162 patients with benign parathyroid lesions hospitalized in PUMCH during the same period were randomly selected according to the ratio of 2:1 to APA cases, including 135 parathyroid adenoma (PA) and 27 parathyroid hyperplasia (PH) patients. All cases included in this study met the criteria for PHPT and were confirmed by histopathology after surgery.

Diagnosis of PHPT was defined biochemically as increased serum calcium (SCa) or albumin-corrected calcium (CCa, CCa = SCa + [0.02 × (40 − albumin)]) level (>2.70 mmol/L) and/or serum ionized calcium (iCa) level (>1.28 mmol/L) combined with unsuppressed PTH level without other causes of hypercalcemia (1).

Histopathology diagnoses were confirmed by the pathologists in our institution. PC was diagnosed on the basis of finding lesions with vascular or perineural invasion, capsular penetration, and/or documented metastases (12). The diagnostic criterion for APA was finding parathyroid tumors partially sharing some of the atypical features in PC, but not enough histopathological criteria for diagnosing PC (6, 12). For patients with multi-glandular involvement, if the pathological findings were different for different lesions, the more malignant areas were chosen for diagnosis.

This study was approved by the Ethics Committee of PUMCH and conducted according to the principles in the Declaration of Helsinki. Written informed consent was obtained from the subjects or from the parents for subjects ≤18 years for genetic analysis.

### 2.2 Clinical and laboratory investigation

The clinical data were retrospectively obtained from medical records reserved at PUMCH, including demographics, clinical manifestations (e.g., bone involvement, urinary system damage, gastrointestinal symptoms, pancreatitis and hypercalcemia crisis), pre-operative biochemical indices, radiographic findings, pathology, follow-up and outcomes (postoperative recurrence and metastasis). Bone involvement included pathological fracture, osteomalacia, and the typical X-ray features of bone resorption (such as subperiosteal absorption and osteitis fibrosa cystica). The definition of asymptomatic PHPT was hyperparathyroidism lacking traditionally specific symptoms or signs associated with hypercalcemia or PTH excess (17). Hypercalcemia crisis was defined as a SCa level of ≥3.5 mmol/L, and usually associated with acute signs and symptoms of hypercalcemia (18). Recurrence was defined as hypercalcemia with high or inappropriately normal serum parathyroid hormone (PTH) level recurring after a disease-free period of at least 6 months after parathyroid operation (19).

Bone mineral density (BMD) was measured by dual-energy X-ray absorptiometry (DXA; GE-Lunar, USA) at the femoral neck (FN, CV: 1.73%) and lumbar spine (L2-L4, CV: 1.70%). Z-scores and T-scores of BMD were calculated using database of normal subjects in our institution (20). Urolithiasis or renal calcification was assessed using ultrasound application. Ultrasonography and 99m-sestamibi-scintigraphy (MIBI) were conducted in all PHPT patients for pre-operative localization.

The serum and urinary biochemical indices were measured in the Department of the Clinical Laboratory of PUMCH. iCa level was measured with a blood-gas analyzer radiometer (ABL800 FLEX; Denmark). SCa, phosphorus (P), alkaline phosphatase (ALP), and 24h urinary calcium (24hUCa) were measured with the Beckman Automatic Biochemical Analyzer (AU5800; Beckman Coulter). The serum PTH level was measured by chemiluminescence (ADVIA Centaur; Siemens, Germany). Serum 25-dihydroxyvitamin D (25OHD) value was measured using an electrochemiluminescence immunoassay (e601; Roche Cobas, Germany).

### 2.3 DNA isolation and gene-mutation analysis

Genomic DNA was extracted from the peripheral blood lymphocytes of 69 patients from PC and APA groups, using the QIAamp Blood DNA Kit (Qiagen; Hilden, Germany) according to the manufacturer’s protocol. A custom-designed gene panel was conducted by Nimblegen SeqCap EZ system (Novogene, Beijing, China) to capture all exons and 10-basepairs (bps) flanking intron sequences of the eight candidate genes for PHPT, i.e., *GCM2, MEN1, RET, CDKN1B, CASR, HRPT2/CDC73, GNA11*, and *AP2S1*. The Targeted Next-Generation Sequencing (NGS) was performed at Novogene Corporation (Beijing, China) and sequenced on the HiSeq 2500 Sequencing System (Illumina, San Diego, CA, USA) as previously described (21). The coverage of target regions of all samples was > 99.9%. The average sequencing depth on target of all samples was > 700×.

All coding exons and exon-intron boundaries of the *CDC73/HRPT2*, *GCM2*, *MEN1, CDKN1B* and *CASR* genes and exons 8, 10-11 and 13-16 of the *RET* gene were amplified *via* polymerase chain reaction (PCR), followed by Sanger sequencing which was performed as previously prescribed (22–26). For those patients with no *CDC73* gene mutation as detected by routine PCR, multiplex ligation-dependent probe amplification (MLPA) was further performed to screen for large deletions in the *CDC73* gene.

In order to uncover genotype-phenotype correlation, the PC and APA patients who had accepted genetic analysis were each divided into two subgroups according to whether they carried nonsynonymous genetic variations or not. Subjects in subgroup 1 and subgroup A carried missense, nonsense, frameshift, gross deletion, and splice site variations. Subjects in subgroup 2 and subgroup B carried synonymous or no variations. Subgroup 1 (A) was compared with subgroup 2 (B) in aspect of clinical characteristics and follow-up outcomes.

### 2.4 Statistical analysis

Statistical analysis of original data was conducted using SPSS Statistics 26.0 (Chicago, Illinois, USA). Normality of distributions was checked. Results were described as percentages for categorical variables, mean ± standard deviation (SD) for normally distributed variables, and median and 25^th^ and 75^th^ interquartile ranges (Q25, Q75) for non-normally distributed variables.

Between-group differences were analyzed using One-Way ANOVA for normally distributed variables and Kruskal-Wallis H Test for non-normally distributed variables. Categorical variables were compared by Pearson chi-square test or Fisher’s exact test, as appropriate. For all analyses, P-value <.05 was considered to be statistically significant.

## 3 Results

### 3.1 Demographics and clinical characteristics

A total of 320 subjects were included in this study, containing 79 cases pathologically diagnosed with APA, 79 cases with PC, 162 cases with benign neoplasms consisting of 135 PA and 27 PH. Comparisons of the demographics, clinical manifestations, biochemical indices, radiographic and densitometric data of the APA, PC and benign groups are shown in **Table 1**.

**Table 1:** Demographics, clinical presentation, biochemical and densitomatric parameters of patients with pathologically confirmed primary hyperparathyroidism.

Parameters		Benign Lesions		Atypical Adenoma		Carcinoma	*P* Values		
	n	value, (%)	n	value, (%)	n	value, (%)	*P1*	*P2*	*P3*
Onset Age (yr)	162	52.0 ± 14.3	79	46.9 ± 17.1	79	45.7 ± 13.8	**0.013**	**0.002**	0.635
Sex									
Male	162	48 (29.6)	79	22 (27.8)	79	48 (60.8)	0.707	**0.000**	**0.000**
Female	162	114 (70.4)	79	57 (72.2)	79	31 (39.2)			
Asymptomatic PHPT	162	53 (32.7)	79	13 (16.5)	79	4 (5.1)	**0.011**	**0.000**	**0.019**
Bone Involvement	162	62 (38.3)	79	28 (35.4)	79	49 (62.0)	0.776	**0.001**	**0.002**
Fragility fractures	162	23 (14.2)	79	11 (13.9)	79	18 (22.8)	0.186		
Rickets/Osteomalacia	162	16 (9.9)	79	4 (5.1)	79	11(13.9)	0.175		
Typical changes in X-rays	162	47 (29.0)	79	24 (30.4)	79	38 (48.1)	0.733	**0.003**	**0.026**
Urolithiasis/Renal Calcification	162	48 (29.6)	79	45 (57.0)	79	54 (68.4)	**0.000**	**0.000**	0.121
Gastrointestinal symptoms	162	26 (16.0)	79	14(17.7)	79	33 (41.8)	0.680	**0.000**	**0.001**
Pancreatitis	162	2 (1.2)	79	3 (3.8)	79	3 (3.8)	0.327		
Hypercalcemic Crisis	162	15 (9.3)	79	18 (22.8)	79	42 (53.2)	**0.003**	**0.000**	**0.000**
Serum iPTH (pg/ml)	162	233.6 (133.8~605.2)	77	593.0 (216.4~1149.0)	78	1128.4 (506.7~1691.8)	**0.000**	**0.000**	**0.013**
Serum total calcium (mmol/L)	162	2.83 ± 0.26	77	3.02 ± 0.44	78	3.39 ± 0.56	**0.003**	**0.000**	**0.000**
Serum ionized calcium (mmol/L)	132	1.42 ± 0.24	69	1.48 ± 0.18	57	1.72 ± 0.32	0.162	**0.000**	**0.000**
Serum phosphorus (mmol/L)	160	0.80 ± 0.20	77	0.71 ± 0.18	76	0.64 ± 0.20	**0.000**	**0.000**	0.061
Serum ALP (U/L)	132	122 (94~196)	71	187 (112~347)	69	367 (145~680)	**0.013**	**0.000**	**0.024**
Serum 25OHD (ng/mL)	100	13.1 (9.0~17.6)	69	9.8 (6.8~12.9)	36	11.3 (8.2~17.6)	0.531	0.831	0.209
24hUCa (mmol/d)	145	8.0 (5.3~10.8)	70	8.8 (5.8~12.2)	55	10.3 (6.7~15.8)	0.366	**0.017**	0.717
LS BMD (Z-score)	45	-0.451 ± 1.378	40	-1.127 ± 1.532	32	-1.056 ± 1.531	**0.037**	0.079	0.839
FN BMD (Z-score)	48	-0.475 ± 1.082	46	-1.446 ± 1.330	35	-1.037 ± 1.352	**0.000**	**0.045**	0.147
LS BMD (T-score)	51	-1.151 ± 1.691	43	-1.747 ± 2.051	37	-1.768 ± 1.584	0.111	0.113	0.958
FN BMD (T-score)	54	-1.317 ± 1.228	47	-2.013 ± 1.286	39	-1.726 ± 1.424	**0.008**	0.138	0.311

Normally distributed variables are expressed as mean ± SD; non-normally distributed variables are expressed as median (interquartile range).

P-values <0.05 are shown in bold.

P1, P-value of comparison between benign lesions and APA; P2, P-value of comparison between benign lesions and PC; P3, P-value of comparison between APA and PC.

iPTH, intact parathyroid hormone; ALP, alkaline phosphatase; 25OHD, 25-hydroxy vitamin D; TCa, total calcium; iCa, ionized calcium; P, phosphorous; 24hUCa, 24h urinary calcium; BMD, bone mineral density; LS, lumbar spine; FN, femoral neck.

Normal reference ranges for indexes: serum TCa: (2.13-2.70) mmol/L; serum iCa: (1.08-1.28) mmol/L; serum P (mmol/L): 11-18 y: 0.81-1.53, >18 y: 0.81-1.45; serum iPTH (13–65):pg/mL; serum 25OHD: >30 ng/mL.

The onset ages of PHPT in the APA and PC groups were not different, while both of them were younger than that of the benign group (46.9 ± 17.1 and 45.7 ± 13.8 vs. 52.0 ± 14.3 yrs). There were 48 males (60.8%) and 31 females (39.2%) in PC patients, with a male to female ratio of 1.55:1. In contrast, females accounted for the majority of both APA (72.2%) and benign lesions group (70.4%), with the female to male ratio being 2.59:1 and 2.38:1, respectively.

Bone involvement, typical X-ray features of PHPT and gastrointestinal symptoms occurred in 28 (35.4%), 24 (30.4%) and 14 (17.7%) patients in the APA group, resembling those of the benign group and significantly less than the PC group (**Table 1**). Urolithiasis/renal calcification was less in patients with benign lesions compared with APA and PC patients (29.6% vs. 57.0% and 68.4%, P<0.001), while no significant difference of that was found in the last two groups. In contrast with the benign lesions group, the proportion of hypercalcemic crisis was significantly higher and the percentage of asymptomatic PHPT was much lower in the PC group [53.2% vs. 9.3% (P=0.000) and 5.1% vs. 32.7% (P=0.000)], while these two proportions of the APA group (22.8% and 16.5%, respectively) lay exactly right between those of the benign lesions and the PC group (P<0.05). The mean serum total calcium and median PTH levels were 3.02 ± 0.44mmol/L and 593.0pg/ml in the APA group. Significant between-group differences were found in serum PTH, serum calcium and ALP levels, with the highest levels being in the PC group and the second being in the APA group (p<0.05). Patients of PC had the lowest level of serum phosphorus and the highest level of 24-hours urinary calcium among the three groups. Z scores at both femoral neck and lumbar spine (L2-L4) of APA and PC patients were lower in contrast to those of patients with benign neoplasms. No remarkable distinctions were found in other clinical, biochemical and densitometric parameters of the three groups (**Table 1**).

### 3.2 Surgery outcomes and follow-up

Details regarding the operations and follow-ups are shown in **Table 2**. The PC group had a significantly higher proportion of parathyroidectomies (PTXs) combined with thyroid lobectomy and central neck dissect (CND) when compared with the benign group, and the APA group was in between. According to the preoperative localization and intra-operative exploration, the majority of the patients in all three groups had a single lesion. The patients with PC had the largest size of parathyroid tumor, with that of APA being the second largest (3.18 ± 1.06 and 2.77 ± 1.03 cm, respectively). Remission was defined as postoperative normalization of serum calcium and PTH levels during the whole follow-up (at least 6 months). No remission (i.e., persistence) was defined as PHPT persistent within 6 months after surgery. Recurrence meant that PHPT occurred again after surgery for at least 6 months.

**Table 2:** Surgery-related variables and outcomes of patients with pathologically confirmed primary hyperparathyroidism.

Parameters	Benign Lesions		Atypical Adenoma		Carcinoma		*P* Values		
	n, (%)	Value	n, (%)	Value	n, (%)	Value	*P1*	*P2*	*P3*
Type of first operation for PC/APN									
PTx	149 (92.0)		55 (69.6)		36 (45.6)		**0.000**	**0.000**	**0.017**
PTx and lobectomy	7 (4.3)		7 (8.9)		29 (36.7)				
PTx, lobectomy and CND	5 (3.1)		13 (16.5)		14 (17.7)				
Thymectomy	1 (0.6)		4 (5.1)		0 (0)				
Number of parathyroid glands in pathology report									
1	145 (89.5)		70 (88.6)		74 (93.7)		0.496		
2	16 (9.9)		8 (10.1)		5 (6.3)				
≥3	1 (0.6)		1 (1.3)		0 (0)				
Size of parathyroid gland (cm)*	123	2.05 ± 1.05	73	2.77 ± 1.03	49	3.18 ± 1.06	**0.000**	**0.000**	**0.033**
Total number of cervical operations for PC/APN									
1	87 (53.7)		59 (74.7)		14 (17.7)		0.854	**0.000**	**0.000**
2	3 (1.9)		3 (3.9)		20 (25.6)				
≥3	2 (1.2)		1 (1.3)		26 (33.3)				
NA	70 (43.2)		16 (20.8)		19 (24.4)				
Follow-up after the initial surgery (months)*	162	62.7 ± 66.4	63	44.2 ± 28.1	62	83.7 ± 65.2	0.051	0.131	**0.000**
Recurrence									
No recurrence	157 (96.9)		59 (74.7)		10 (12.7)		0.271	**0.000**	**0.000**
No remission/Loco-regional	5 (3.1)		4 (5.1)		25 (31.6)		0.271	**0.000**	**0.000**
Metastasis	0 (0)		0 (0)		27 (34.2)				
Loss of follow	0 (0)		16 (20.3)		17 (21.5)				
Metastasis location									
Lymph nodes	/		/		15 (55.6)				
Lung	/		/		19 (70.4)				
Bone	/		/		2 (7.4)				
Liver	/		/		1 (3.7)				

Data are expressed as mean ± SD or as percentage.

P-values <0.05 are shown in bold.

P1, P-value of comparison between benign lesions and APA; P2, P-value of comparison between benign lesions and PC; P3, P-value of comparison between APA and PC.

* n: the number of the cases measured for the index. /, not available.

PC, parathyroid carcinoma; APN, atypical parathyroid neoplasm; PTx, parathyroidectomy; CND, central neck dissect.

NA, not available.

The mean periods of follow-up after the initial parathyroid surgery of the PC, APA and benign lesions groups were 83.7 ± 65.2, 44.2 ± 28.1 and 62.7 ± 66.4 months, respectively. Meanwhile, the number of patients loss to follow-up in the APA and PC were 16 (20.3%) and 17 (21.5%), respectively. Patients with PC had a significantly higher rate of cervical reoperations than the APA and benign groups (p = 0.000). Four patients in the PC group and one in the APA did not obtain remission after their first operations. The regional recurrence occurred in 5 (3.1%), 3 (3.8%) and 21 (26.6%) cases of the benign lesions, APA and PC groups, respectively (P=0.000). A total of 27 PC patients developed distant metastasis. The metastasis locations contained lymph nodes, lung, bone and liver, with the numbers of cases being 15 (55.6%), 19 (70.4%), 2 (7.4%) and 1 (3.7%), respectively. Furthermore, 5 and 10 cases from the PC group had been diagnosed as atypical adenoma and benign adenoma, respectively, according to the histopathology at their first operations. And two APA patients had been diagnosed with benign adenoma by first surgery.

### 3.3 Genetic analysis

Among the 38 PC patients and 31 APA patients who agreed to genetic analysis, except for one PC case carrying single nucleotide synonymous variant of *AP2S1* gene, 8 APA patients (25.8%, 8/31) and 17 PC patients (44.7%, 17/38) were found to carry germline nonsynonymous rare variants of the candidate genes, including 14 *CDC73*, 4 *GCM2*, 3 *MEN1*, 2 *RET*, 1 *GNA11* and 1 *CDKN1B* gene variants (**Table 3**). No alterations in *CASR* gene were found. The *CDC73* gene accounted for the majority of all altered genes in both PC (64.7%, 11/17) and APA groups (37.5%, 3/8). Among all the 14 *CDC73* gene mutations, there were seven frameshift mutations (50.0%), five nonsense mutations (35.7%), one splice site mutations (7.1%), and one gross deletion mutation (7.1%). Mutations in exon 7 were observed more frequently (57.1%, 8/14) than those in other exons, but hot spot mutation region was not found. One PC patient and one APA patient were confirmed to have family history of PHPT. The overall recurrence/no remission/metastasis rate was as high as 72.0% (18/25) among the nonsynonymous variants carriers regardless of whether or not they had family history of PHPT, while there were only two APA patients (25.0%, 2/8) among them and all the rest were PC patients (43.2%, 16/37). Only two of the four APA patients presenting recurrence or no remission accepted genetic analysis and carried a nonsense mutation in exon 7 of the *CDC73* gene and a missense *MEN1* variants.

**Table 3:** Germline variants of candidate genes for primary hyperparathyroidism in studied APA and PC patients.

Case	Pathology	Family History	Onset Age	Sex	Gene	Nucleotide Alteration	Variation Type	Location	ACMG	Outcome	Reference
1	PC	NO	58	M	*CDC73*	c.687_688 delAG	Frameshift	Exon 7	P	Recurrence	Howell et al., 2003 (27)
2	PC	NO	29	M	*CDC73*	c.664 C>T	Nonsense	Exon 7	P	Recurrence	Shattuck et al., 2003 (28)
3	PC	NO	39	M	*CDC73*	c.162 C>G	Nonsense	Exon 2	P	Metastasis	Howell et al., 2003 (29)
4	PC	NO	60	M	*CDC73*	c.626_629 delAACA	Frameshift	Exon 7	P	Metastasis	Wang et al., 2012 (30)
5	PC	NO	52	M	*CDC73*	c.260_261 delGA	Frameshift	Exon 3	P	Metastasis	Wang et al., 2012 (30)
6	PC	NO	36	M	*CDC73*	c.685_688 delAGAG	Frameshift	Exon 7	P	Metastasis	Guarnieri et al., 2006 (31)
7	PC	NO	27	M	*CDC73*	c.664 C>T	Nonsense	Exon 7	P	No remission	Shattuck et al., 2003 (28)
8	PC	NO	55	M	*CDC73*	c.34_35 insCT	Frameshift	Exon 1	P	Recurrence	Wang et al., 2012 (30)
9	PC	NO	35	M	*CDC73*	c.729+1 G>A	Splice site	Exon 7	P	Recurrence	Novel
10	PC	YES	28	F	*CDC73*	Del exons 1-6	Gross deletion	Exons 1–6	P	Recurrence	Yang et al., 2022 (32)
11	PC	NO	39	F	*CDC73*	c.40 C>T	Nonsense	Exon 1	P	Metastasis	Kong et al., 2014 (23)
12	PC	NO	15	M	*GCM2*	c.1247 A>G	Missense	Exon 5	VUS	Recurrence	Song et al., 2020 (26)
13	PC	NO	60	M	*GCM2*	c.1162 A>G	Missense	Exon 5	P	No remission	Song et al., 2020 (26)
14	PC	NO	64	M	*GCM2*	c.1162 A>G	Missense	Exon 5	P	No recurrence	Song et al., 2020 (26)
15	PC	NO	49	M	*MEN1*	c.549 G>C	Missense	Exon 3	VUS	No remission	Lee et al., 2014 (33)
16	PC	NO	47	M	*CDKN1B*	c.225 G>C	Missense	Exon 1	VUS	No remission	Novel
17	PC	NO	29	M	*GNA11*	c.321+9 G>A	Splice site	Exon 2	VUS	Recurrence	Novel
18	PC	NO	24	F	*AP2S1*	c.309 C>T	Synonymous	Exon 3	VUS	Metastasis	Novel
19	APA	NO	11	M	*CDC73*	c.570 delG	Frameshift	Exon 7	P	No recurrence	Kong et al., 2014 (23)
20	APA	NO	15	F	*CDC73*	c.1303-1304 insA	Frameshift	Exon 14	P	No recurrence	Wang et al., 2017 (34)
21	APA	NO	18	F	*CDC73*	c.700 C>T	Nonsense	Exon 7	P	Recurrence	Cetani et al., (35)
22	APA	YES	51	F	*MEN1*	c.917 T>G	Missense	Exon 6	VUS	No remission	Song et al., 2020 (36)
23	APA	NO	52	F	*MEN1*	c.431 T>C	Missense	Exon 2	VUS	No recurrence	Song et al., 2020 (36)
24	APA	NO	53	M	*GCM2*	c.1144 G>A	Missense	Exon 5	P	No recurrence	Song et al., 2020 (26)
25	APA	NO	30	M	*RET*	c.874 G>A	Missense	Exon 5	VUS	No recurrence	Novel
26	APA	NO	52	F	*RET*	c.833 C>A	Missense	Exon 4	VUS	No recurrence	Novel

PC, parathyroid carcinoma; APN, atypical parathyroid neoplasm; M, male; F, female.

According to the American College of Medical Genetics and Genomics (ACMG) Standards and Guidelines (37), variants were classified into five categories: pathogenic (P), likely pathogenic (LP), variants of uncertain clinical significance (VUS), likely benign, and benign.

### 3.4 Genotype-phenotype correlations

The 38 PC patients and 31 APA patients were each divided into two subgroups, to assess the influence of nonsynonymous variants of those PHPT-related genes on the patients’ clinical characteristics and prognosis (**Table 4**). The subgroup 1 of the APA subjects, consisting of 8 patients having nonsynonymous variants, showed an earlier onset age for PHPT in comparison with subgroup 2, that contained 23 patients with no variations or synonymous variants (28.5 ± 16.9 vs. 48.1 ± 17.7 yrs, P<0.01). As for the clinical, biochemical and radiological characteristics, neither the APA group nor the PC group presented statistically significant distinctions between their two subgroups, except for a higher serum ALP concentration of the APA subgroup 1 and more elevated serum iPTH level of the PC subgroup b. The recurrence rate of the APA subgroup 1 had reached 25% (2/8) but it turned out to have no statistical significance compared to that of the subgroup 2. Those PC patients with nonsynonymous variations (i.e., the PC subgroup A) showed a higher proportion of no remission and regional reoccurrence.

**Table 4:** Comparison of clinical characteristics and outcomes between patients with nonsynonymous variations and without such variations.

Parameters	Atypical Adenoma		Carcinoma		*P* Values	
	subgroup 1*	subgroup 2#	subgroup A*	subgroup B#	*P1*	*P2*
	n=8	n=23	n=17	n=21		
Onset Age (yr)	28.5 ± 16.9	48.1 ± 17.7	40.9 ± 13.5	42.1 ± 12.4	**0.006**	0.801
Sex						
Male	3 (37.5)	7 (30.4)	15 (88.2)	13 (61.9)	1.000	0.136
Female	5 (62.5)	16 (69.6)	2 (11.8)	8 (38.1)		
Asymptomatic PHPT	0(0)	4 (17.4)	0(0)	0(0)	0.553	
Bone Involvement	2 (33.3)	9 (39.1)	10 (58.8)	15 (71.4)	1.000	0.489
Fragility fractures	2 (33.3)	4 (17.4)	5 (31.3)	6 (28.6)	0.575	1.000
Rickets/Osteomalacia	0 (0)	3 (13.0)	4 (25.0)	3 (14.3)	1.000	0.437
Typical changes in X-rays	1 (16.7)	7 (30.4)	8 (50.0)	9 (42.9)	0.647	0.746
Urolithiasis/Renal Calcification	7 (87.5)	10 (43.5)	15 (88.2)	15 (71.4)	0.169	0.423
Gastrointestinal symptoms	0 (0)	7 (30.4)	7 (43.8)	8 (38.1)	0.289	0.749
Pancreatitis	0 (0)	0 (0)	0(0)	1 (4.8)		1.000
Hypercalcemic Crisis	0 (0)	9 (39.1)	8(50.0)	13 (61.9)	0.137	0.519
Serum iPTH (pg/ml)	1010.8 (263.1~2193.4)	687.0 (159.9~1296.1)	852.5 (417.5~1115.4)	1386.7 (1180.5~1885.5)	0.389	**0.000**
Serum total calcium (mmol/L)	3.03 ± 0.31	3.05 ± 0.46	3.47 ± 0.59	3.49 ± 0.52	0.931	0.901
Serum ionized calcium (mmol/L)	1.47 ± 0.21	1.50 ± 0.19	1.68 ± 0.26	1.69 ± 0.27	0.806	0.881
Serum phosphorus (mmol/L)	0.66 ± 0.18	0.64 ± 0.23	0.52 ± 0.19	0.69 ± 0.23	0.890	0.057
Serum ALP (U/L)	1117 (292~2132)	197 (134~277)	397 (155~847)	386 (164~678)	**0.011**	0.944
Serum 25OHD (ng/mL)	12.1 (6.6~14.9)	10.4 (6.5~15.6)	11.3 (6.2~19.1)	11.4 (7.9~18.6)	0.946	0.851
24hUCa(mmol/d)	6.5(4.2~12.1)	9.6(4.6~13.1)	9.6(7.2~19.6)	9.1(6.8~13.2)	0.454	0.437
LS BMD (Z-score)	0.167 ± 1.750	-1.210 ± 1.583	-0.680 ± 1.548	-1.054 ± 1.492	0.162	0.522
FN BMD (Z-score)	-0.275 ± 1.069	-1.524 ± 1.171	-1.173 ± 1.348	-1.009 ± 1.160	0.064	0.835
LS BMD (T-score)	-1.167 ± 0.808	-2.105 ± 2.515	-0.930 ± 1.644	-1.825 ± 1.548	0.136	0.354
FN BMD (T-score)	-0.867 ± 0.611	-2.282 ± 1.307	-1.518 ± 1.413	-1.900 ± 1.208	0.081	0.587
Number of parathyroid glands in pathology report					0.077	0.399
1	4 (50.0)	19 (82.6)	15 (88.2)	20 (95.2)		
2	3 (37.5)	4 (17.4)	2 (11.8)	1 (4.8)		
≥3	1 (12.5)	0 (0)	0 (0)	0 (0)		
Size of parathyroid gland (cm)	2.86 ± 1.04	2.73 ± 0.67	2.95 ± 0.80	3.63 ± 1.38	0.783	0.130
Follow-up after the initial surgery (months)#	62.3 ± 30.5	37.6 ± 21.8	79.0 ± 59.0	98.1 ± 69.2	0.318	0.471
Outcome						
Remission	6 (75.0)	18 (78.3)	1 (5.9)	4 (19.0)	1.000	0.236
No remission/Loco-regional recurrence	2(25.0)	0(0)	11(64.7)	3 (14.3)	0.086	**0.038**
Metastasis	0(0)	0(0)	5(29.4)	11 (52.4)		1.000
Loss of follow	0(0)	5 (21.7)	0(0)	3 (14.3)		

Normally distributed variables are expressed as mean ± SD; non-normally distributed variables are expressed as median (interquartile range).

P-values <0.05 are shown in bold.

*Subgroups with nonsynonymous variations.

#Subgroups with synonymous or no variations.

P1, P-value of comparison between subgroup 1 and 2; P2, P-value of comparison between subgroup A and B.

## 4 Discussion

Atypical adenoma and carcinoma only account for a very small proportion of primary hyperparathyroidism. The clinical and genetic data are still limited. Here we report the largest single-center cohort of patients with APA (n=79) followed by a mean time of approximately 3.7 years, comparing the clinical characteristics as well as outcomes after surgeries with patients with PC (n=79) and benign parathyroid tumors (n=162). Furthermore, genetic screening for candidate genes of PHPT were performed in about half of APA and PC patients to provide a genotype-phenotype analysis.

The mean onset age of APA was comparable to that of PC in our study, while both of them were 5-6 years younger than that of the benign controls. A review lately summed up 672 APA patients from available research and found the mean age at diagnosis of APA being 43.3 ± 18.9 years, which was, as in PC, a decade earlier than the typical age of benign disease (6). Besides, this review also provided a female to male ratio in APA patients as about 1.5: 1 (6), relatively lower than that in our cohort (2.6: 1) and both lower than that reported in the benign counterpart, who showed a female predominance with a ratio of 3–4: 1 (6, 15, 38, 39). As for PC, there was no gender preference (15, 40).

Our study demonstrated that APA had a relatively benign clinical behavior. Similar to the benign group, the APA patients showed less bone involvement and typical changes in X-rays (bone resorption) than the PC group. But the results of BMD suggested that APA might be more similar to PC as for osteoporosis. For the incidence of urolithiasis, the APA patients were alike the PC patients. But for the gastrointestinal symptoms and asymptomatic PHPT, the APA patients behaved closer to the benign controls. In the present study, the APA group had a ratio of bone involvement within the range reported by the previous literatures from western countries as 35.4% vs. 20.0~67.9%, and a relatively higher ratio of kidney manifestations as 57.0% vs. 32.1~50.0% (6, 7, 41–43). The two largest cohorts in existing studies, reported by Saponaro et al. (7) and Schneider et al. (10), contained 58 APA patients with 57 PA controls and 68 APA with 15 PC controls, respectively. Saponaro et al. (7) reported that the ratio of nephrolithiasis of the whole APA group was 34.5% (20/58), being higher than the PA controls (28.1%, 16/57). Schneider et al. (10) found that bone alterations of APA was significantly less than that of PC (34% vs. 60%, *P*=0.01), but their ratios of renal stones were equivalent (34% vs. 20%, *P=*0.37). Although our results suggested that the APA patients had more benign clinical presentations than PC, none of these symptoms or signs were specific for them.

The levels of serum calcium and PTH of our benign lesions and PC groups were comparable to those reported in the largest cohort of PHPT in Chinese people (39). Moreover, our study suggested that the biochemical profile of APA was less severe than PC’s though not as mild as that of benign neoplasms. The APA group had serum calcium level, serum PTH level and proportion of hypercalcemic crisis all higher than those of benign lesions but lower than those of PC group. According to available data, the ranges of median serum PTH and mean SCa levels were 133~1833ng/L and 11.7~15.8mg/dL in APA patients (6, 7, 41, 43, 44). A systematic review concluded that patients with APA were characterized by moderate hypercalcemia (mean 13.4 ± 2.4mg/dL) associated with elevated PTH level [median 430 (73–3242) ng/L], 6.6 times above the upper value of normal range (6). Our biochemical results, as well as current data from the literature, had shown an overlap among series of parathyroid typical and atypical adenoma and carcinoma (6). Thus, no reliable biochemical marker could help identifying APA patients carrying the risks of malignant behaviors.

In our study, the recurrence/no remission rate of the APA group, being 3.9%, was significantly lower than that of the PC group and almost the same as the benign group. Four patients in the PC group and one in the APA group did not obtain remission after their first operation, with the former all carrying nonsynonymous variations of candidate genes for PHPT and the latter finally being diagnosed with MEN1. The rate of recurrence/persistence of atypical parathyroid adenomas ranged from 0% to 3.7% in most literatures (6), however, much higher rates were also reported by three studies as 10.3% (6/58) (7), 24% (4/17) (42) and 28.6% (2/7) (45). Our study emphasized that the prognosis of APA was rather better than PC. But it should be taken into account that the mean follow-up of APA patients was 1.3 years shorter than that of PC patients. A relatively high percentage of pathologic diagnosis as adenoma at the first operation in PC (12.7%) and APA (2.5%) patients further confirmed the dilemma of the differentiation between benign and malignant lesions. Besides, a total of 5 PC cases (6.3%) in this study had been misdiagnosed as APA for the pathohistology of their first surgery, suggesting the necessity for APA patients to be continuously monitored. McCoy et al. (46) observed that none of the 51 APA patients recurred over a mean follow-up of 5 years (range, 0.5-18). Nevertheless, Schulte et al. (9) reported that 23.5% (4/17) APA patients developed recurrence over an average follow-up of 107.2 ± 82.7 months, with two of them diagnosed as PC and one suspicious for PC. Christakis et al. (43) provided a 5-year recurrence-free survival rate as 90.91% (95% CI 50.81–98.67) of the APA patients, whose time from initial surgery to first recurrence was longer than that of the PC group, although it did not achieve statistical significance. While it’s known that PC patients should be followed-up lifelong, the duration and frequency of follow-up for APA patients remain to be decided depending on more long-term data.

Germline variations of the candidate genes for PHPT were screened in our study, and the *CDC73* gene was the most frequent mutated gene among all the candidate genes detected not only in PC as expected, but also in APA subjects. The molecular pathogenic mechanisms underlying the origin of atypical adenomas had been investigated only in a few studies until now. Loss-of-function mutations of the *CDC73* gene were the most frequent genetic anomaly for PC, with germline mutations of the *CDC73* gene being presented in one-third of apparently sporadic PC and 90% of HPT-JT patients (28). Oppositely, *CDC73* mutations were very rare in benign neoplasms (27, 47). A latest review of APA summarized the inactivating *CDC73* mutations in various studies, finding 36.5% (23/63) cases carrying germline mutations, including two large deletions spanning exons 1–10 of the gene, most of them being found in familial cases (11 patients with FIHP and 4 with HPT-JT) and 7 in apparently sporadic PHPT (6). Saponaro et al. (7) had screened for the germline *CDC73* mutations in APA patients and found the mutation rate to be four out of 56 (~7%) with two FIHP and two sporadic cases, similar to that displayed in our study (3/31, ~9.7%) and lower than the number concluded in that review. The reason might be the distinction of the proportion of family-related cases. Apparently, the probability of carrying *CDC73* mutations in APA patients was lower than that in PC patients. As mentioned before, two patients with APA and another with PC in our cohort were found out all carrying a missense variation of *MEN1* gene (36). PC occurred very rarely in MEN1-HPT, whose parathyroid lesions were almost exclusively benign (48). Ten APA patients from several studies had been searched for *MEN1* loss-of-function mutations and only one germline mutation (c.253A>T, p.127S) in exon 2 was detected (29, 49–51). Two *GCM2* variants (c.1162A>G and c.1247A>G) detected in our PC patients and one (c.1144 G>A) in this cohort of APA patients were reported in our earlier study (26). The frequency of germline *GCM2* variants in clinically sporadic PHPT cases was less than 5% in almost all populations (26). Existing research suggested that mutations in *GCM2* could increase the risk of familial or sporadic PHPT (26, 52, 53) and had a tendency for malignancy (26, 53–55), indicating that it might be beneficial for PC and APA patients without CDC73 mutations to screen for GCM2 mutations.

The APA subjects, carrying nonsynonymous variants, showed earlier onset age but no other distinctions for clinical characteristics and outcomes, in comparison with the other subgroup without gene alterations. Although with no statistical significance (*P*=0.086), two out of 8 (25%) APA cases with nonsynonymous variants developed recurrence or no remission, whereas all cases of no-variation subgroup showed no relapse. As we expected, gene variations were associated with more recurrence/no remission of PHPT in parathyroid carcinoma, although they seemed to have not so much influence on the pre-surgical clinical characteristics of PC. These results required further demonstration due to the limited sample size. Until now, only a few studies have investigated the effects of molecular alterations on APA patients’ clinical outcome and concentrated on CDC73 gene. Cetani et al. (6) concluded that after a time of follow-up ranged from 23 to 252 months (mean 110 ± 71 months), 42% of APA patients carrying germline CDC73 mutations had recurrence/persistence of disease, while 95% CDC73 mutation-negative patients were cured by operation. The study of Saponaro et al. (7)showed that 50% (2/4) APA patients harboring *CDC73* germline mutations and 7.7% (4/52) of those without *CDC73* mutation had persistent/recurrent disease. From these facts we could draw a conclusion that closer and prolonged surveillance should be beneficial to APA patients carrying germline variations, especially of CDC73 gene. Hence, genetic analysis is probably useful and recommended for APA patients.

The current study has several limitations. First, it is a retrospective study and data missing is unavoidable. About 20% subjects were lost to follow-up. Second, the sample size for genetic screening is restricted. However, considering the rarity of APA and PC, these results are still valuable for clinicians and investigators. Third, somatic mutation detection of parathyroid lesions was not conducted, due to lacking of the surgical specimens.

In summary, our study shows that patients with APA present clinical and biochemical characteristics much less severe than PC and resembling the benign neoplasms, as well as having a relatively good prognosis. The molecular signature of APA remains unclear and the germline *CDC73* mutations appears to be the most common abnormality in this series. Patients with APA carrying germline gene variations may have an earlier onset and greater possibility of recurrence for PHPT, probably needing more positive follow-up strategies. More evidence and longer follow-up studies are necessary.

## Data availability statement

The datasets presented in this study can be found in online repositories. The names of the repository/repositories and accession number(s) can be found in the article/**Supplementary Material**.

## Ethics statement

The studies involving human participants were reviewed and approved by The Institutional Review Board of Peking Union Medical College Hospital (PUMCH). Written informed consent to participate in this study was provided by the participants’ legal guardian/next of kin.

## Author contributions

OW, XX, MN, YJ, ML, and WX contributed to conception and design of the study. YC and AS performed research, analyzed data, and contributed to the discussion and writing of the manuscript. OW and XX contributed to the discussion and edition of the manuscript. All authors contributed to the article and approved the submitted version.
